# One-dimensional polymer-derived ceramic nanowires with electrocatalytically active metallic silicide tips as cathode catalysts for Zn–air batteries†

**DOI:** 10.1039/d1ra05688c

**Published:** 2021-12-13

**Authors:** Prabu Moni, Marek Mooste, Kaido Tammeveski, Kurosch Rezwan, Michaela Wilhelm

**Affiliations:** University of Bremen, Advanced Ceramics Am Biologischen Garten 2, IW3 Bremen 28359 Germany mwilhelm@uni-bremen.de +49 421 218 64932 +49 421 218 64944; Institute of Chemistry, University of Tartu Ravila 14a 50411 Tartu Estonia; University of Bremen, MAPEX Center for Materials and Processes Bibliothekstraße 1 28359 Bremen Germany

## Abstract

New metallic nickel/cobalt/iron silicide droplets at the tips of polymer-derived ceramic (PDC) nanowires have been identified as stable and efficient cathode catalysts for Zn–air batteries. The as-prepared catalyst having a unique one-dimensional (1D) PDC nanowire structure with the presence of metallic silicide tips of 1D-PDC plays a crucial role in facilitating oxygen reduction/evolution reaction kinetics. The Zn–air battery was designed using Ni/PDC, Co/PDC and Fe/PDC as air electrode catalysts. In electrochemical half-cell tests, it was observed that the catalysts have a good bifunctional electrocatalytic activity. The efficiency of the catalysts to function as a cathode catalyst in real-time primary and mechanically rechargeable Zn–air battery configurations was determined. The primary battery testing results revealed that Ni/PDC and Co/PDC exhibited a stable discharge voltage plateau up to 29 h. The Fe/PDC sample, on the other hand, performed up to 23 h with an operating potential of 1.20 V at the discharge current density of 5 mA cm^−2^ after which the battery ceased to perform. The Ni/PDC, Co/PDC, and Fe/PDC cathode catalysts performed galvanostatic 1200 charge–discharge cycles in a mechanically rechargeable secondary Zn–air battery configuration. The results demonstrate that the Ni/PDC, Co/PDC, and Fe/PDC materials serve as excellent and durable bifunctional cathode electrocatalysts for Zn–air batteries.

## Abstract 2

New intermetallic silicide catalysts for Zn–air batteries facilitate ORR/OER kinetics and deliver peak power densities of 59 mW cm^−2^ and 1200 cycles.

## Graphical abstract

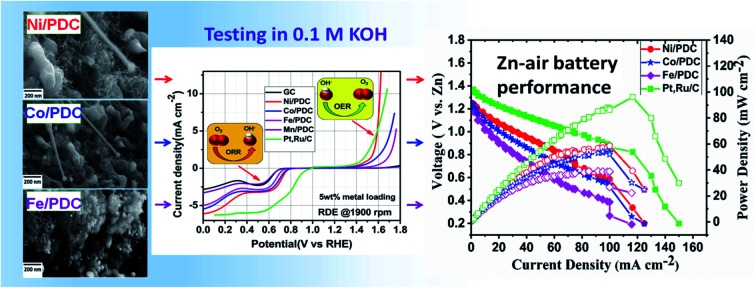

## Introduction

Reversible electrochemical energy storage systems are considered as an up-and-coming energy source and as a replacement to conventional non-renewable energy sources. Electrochemical batteries are an alternative clean energy system and a pragmatic solution to cover the necessary energy demand and preserve the environment from existing noxiousness (due to the CO_2_ emission from fossil fuels). They are considered promising and prodigious because of their simple operation involving redox reactions and higher efficiency.^1,2^ Currently, Li-ion batteries (LIBs) are commercially successful in powering portable electronic devices due to their high coulombic efficiency and long cycling life.^3,4^ However, their progress is not sufficient to satisfy future energy demands, as they do not have a high maximum energy density and have high costs due to the scarcity of lithium metal. Next-generation metal–air batteries are deemed as a breakthrough technology with exceptionally high energy density for power transportation and storage applications. They are characterized by their simple operation, half-open structure, and environmental congeniality.^5–7^ This is why the primary Zn–air battery has already positively conquered the commercial markets of medicine, telecommunication, navigation and railway signal applications. It also satisfies industrial requirements with a high theoretical gravimetric energy density of 1084 W h kg^−1^, minimal cost, and high availability (zinc ranks fourth in metal production quantity). The technical superiority, such as safety and a long cell life, and notably flat charge–discharge voltage in the alkaline environment of the Zn–air battery has attracted attention.^8–10^ Apart from the positive aspects, the slow oxygen reduction reaction (ORR) and oxygen evolution reaction (OER) kinetics of the cathode catalyst is considered to be the core reason for the lower energy density of the Zn–air battery, which impedes the commercialization of the device. The oxygen electro-kinetics of the air electrode play a pivotal role in the resultant efficiency of Zn–air batteries. The sluggish oxygen kinetics of the cathode catalyst begets the Zn–air battery to operate at a large overpotential during discharging (less than 1.65 V), and conversely, a higher potential (more than 1.65 V) is required for charging the cell.^11^ To overcome these practical issues, an efficient bifunctional catalyst to perform fast ORR and OER simultaneously is a necessity.^12–17^ The catalytic performance depends on the composition, structure, porosity, surface area, and electrical conductivity of the material.^18–24^ At this juncture, polymer-derived ceramics (PDC) is a potential candidate for oxygen electrocatalysis due to its flexibility in the synthesis technique to acquire a material of required morphology and structure with unique properties.^25,26^ Silicon-based PDC has already been explored as an anode material for lithium (LIB)/sodium ion battery (NIB) where a LIB made with SiOC material produced a charge capacity of 588 mA h g^−1^ at 1020 cycles, while a NIB showed stable long-term performance over 500 cycles.^27,28^ Vakifahmetoglu *et al.* and Adam *et al.* have already investigated the synthesis of 1D PDC nanowires and catalyst tips of PDC nanowires with the addition of transition metals to preceramic polymers using catalyst-assisted pyrolysis (CAP) method.^29–31^ Recent studies show that ultralow transition metal catalyst with tunable compound exhibits optimized adsorption of the oxygen species (OH^−^, O_2_) and more catalytic sites for both ORR and OER kinetics.^32–35^ In this context, we have recently reported Fe, Ni, Co, and Pt-based metal silicide containing ceramic composites with *in situ* grown carbon nanotubes as efficient bifunctional catalysts towards oxygen in both ORR and OER and their application in anion-exchange membrane fuel cell and Zn–air battery.^36,37^ These results show that the *in situ* grown carbon nanotubes improve the electrical conductivity and formation of intermetallic nickel/cobalt silicide nanocomposite boosting the electrocatalytic activity towards ORR/-OER with relatively low oxygen electrode potential. In this work, we investigate the possibility of preparing 1D PDC nanowires with electrocatalytically active intermetallic silicide tips of PDC nanowires as cathode catalyst for primary and mechanically rechargeable Zn–air battery configurations.

## Experimental section

The catalyst assisted pyrolysis (CAP) method is applied in the synthesis of 1D PDC nanowires.^30,31^ The polymeric silicone resin poly(methyl phenyl silsesquioxane) (Wacker Chemie AG), cross-linking agent (3-aminopropyl)triethoxysilane (APTES, Sigma-Aldrich) and metal salt such as nickel acetylacetonate/cobalt acetylacetonate/iron acetylacetonate/manganese acetylacetonate (Sigma-Aldrich) was dissolved separately in tetrahydrofuran (Sigma-Aldrich) and mixed together under constant stirring. The mixture is then heated in an oil bath to 80 °C under reflux conditions. At 80 °C, the forming agent azodicarbonamide (ADA, Sigma-Aldrich), is added, and the mixture is refluxed and stirred for 24 h. The solvent is removed with the help of a rotary evaporator. Afterwards, the dried samples are cross-linked in a furnace at 120 °C for 3 h in air and pyrolyzed at 1400 °C under N_2_ atmosphere. The samples include X/PDC, where X = Ni, Co, Mn, and Fe are denoted as Ni/PDC, Co/PDC, Mn/PDC and Fe/PDC, respectively. Detailed materials and their composition are presented in Table S1 in ESI.†

X-ray diffraction (XRD) data were collected using a Seifert XRD powder diffractometer (Cu Kα radiation, model 3303, General Electric, USA). Raman spectra were recorded with the help of LabRAM ARAMIS (Horiba Jobin Yvon, 633 nm). The morphological features were analyzed using FESEM (ZEISS Supra 40, Oberkochen, Germany) and FETEM (FEI Titan 80, 300 kV). Energy-dispersive X-ray spectroscopy (EDX) was also performed using FETEM (FEI Titan 80, 300 kV). The Brunauer–Emmett–Teller (BET) surface areas were calculated using N_2_ adsorption/desorption isotherms with a Belsorp-Mini (Bel Japan Inc.). Scanning transmission electron microscopy (STEM) images were acquired by FEI Titan Themis 200 microscope equipped with a Super X detector system for EDX. Catalyst materials were dispersed in ethanol and sonicated. The dispersion was placed on a TEM copper grid with carbon film and dried. For Ni and Si placement detection, the corresponding K-series EDX lines were used.

For the electrochemical experiments, a glassy carbon (GC) electrode with a geometric area (*A*) of 0.196 cm^2^ was used as an underlying substrate. For coating the GC electrode with the catalyst materials, 8 mg of the corresponding PDC catalyst was dispersed in 2 mL of 2-propanol by sonication for 1 h. In the case of Pt, Ru/C (HiSPEC 12100, 50% Pt and 25% Ru; Alfa Aesar) catalyst, 1.33 mg of Pt, Ru/C was dispersed in 2 mL 2-propanol. After the sonication, 5 × 2 μL of the catalyst ink was pipetted onto the polished GC surface. After evaporation of the solvent in air, 0.2 mg cm^−2^ PDC catalyst loading was obtained.^38^ For Pt, Ru/C coated GC electrode, the catalyst loading was 25 μg Pt, Ru cm^−2^. Electrochemical measurements were carried out in Ar-saturated (99.999%, Linde) or O_2_-saturated (99.999%, Linde) 0.1 M KOH (p.a. quality, Merck) using cyclic voltammetry (CV) and linear sweep voltammetry in a rotating disc electrode (RDE) setup. Electrochemical experiments were performed with an Autolab potentiostat/galvanostat PGSTAT128N (Metrohm Autolab, The Netherlands) controlled with Nova 2.1 software. A three-compartment electrochemical cell was used with the catalyst-coated GC electrode as a working electrode, saturated calomel electrode (SCE) as a reference electrode, and Pt wire as a counter electrode. All the potentials presented in this study are referred to the RHE. For the conversion of potential values from *vs.* SCE to *vs.* RHE, the equation *E*_RHE_ = *E*_SCE_ + 0.241 V + 0.059 × pH was used. For the determination of electrochemical double layer capacitance (*C*_dl_), the CV curves were recorded at several potential scan rates (10; 20; 30; 40; 50 mV s^−1^) in the non-faradaic region with a potential window of 200 mV. The following equation was used for the calculation of *C*_dl_:^39^1
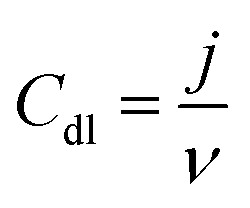
where *j* is the cathodic current density measured at 0.915 V *vs.* RHE on the cyclic voltammogram and *ν* is the corresponding scan rate. For the RDE measurements, an EDI101 rotator and a CTV101 speed control unit (Radiometer) were employed. The following electrode rotation rates (*ω*) were used for RDE experiments: 360, 610, 960, 1600, 1900, 3100, and 4600 rpm. The *j*–*E* curves presented for ORR are recorded in the cathodic direction and the polarization curves for OER in anodic direction.^40^ Both the OER and ORR *j*–*E* curves have been *iR* compensated using the ohmic resistance values obtained with electrochemical impedance spectroscopy.^41^

For the construction of the Zn–air battery, 6 mg of ceramic catalyst and 12 mg of 5% Nafion ionomer solution (Sigma-Aldrich) were dispersed in 0.7 ml of ethanol by sonication for 1 h. The prepared ink was spread on a commercial gas diffusion layer (GDL, SGL DC – 35) by hand brush coating technique with the geometric area (*A*) of 2.25 cm^2^ to obtain the air electrode with a loading of 1 mg cm^−2^. The Zn–air battery experiments were performed with a homemade single-cell Zn–air battery setup controlled with a potentiostat/galvanostat (Bio-Logic, VMP3, France). More details of the homemade zinc–air battery (ZAB) setup were presented in our previous study.^37^ A commercially available zinc metal sheet with a purity of 99% was utilized as an anode. Nickel mesh acted as a current collector. As a separator, a micro-porous polyolefin membrane (Celgard-5550) dipped in the electrolyte (6 M KOH) was used. The cell was assembled by sandwiching the membrane between zinc anode and air breathing cathode.^37^ The specific capacity of prepared ceramic catalysts incorporated Zn–air battery is calculated based on the mass of the consumed zinc metal.

## Result and discussion

**Fig. 1:**
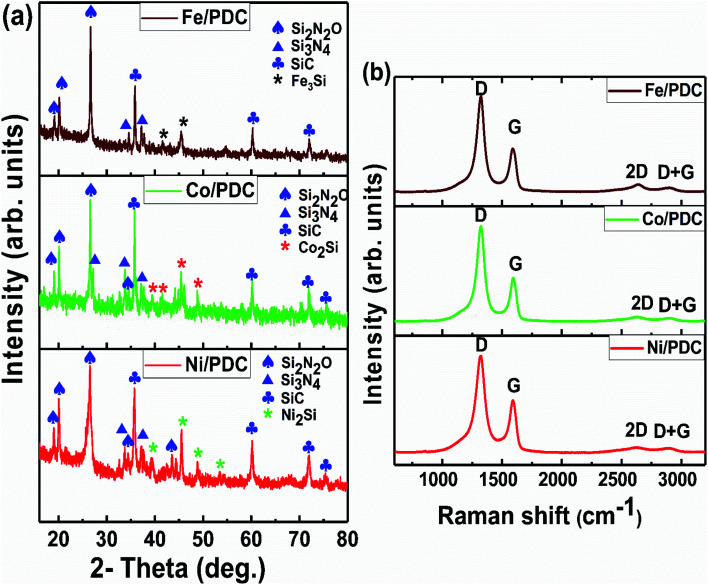
(a) XRD patterns and (b) Raman spectra of the ceramic samples Ni/PDC, Co/PDC, and Fe/PDC.

The powder X-ray diffraction patterns and Raman spectra of PDC samples pyrolyzed at 1400 °C are reported in Fig. 1. The XRD patterns of all ceramic samples clearly show the formation of a well-defined peak of crystalline SiC (JCPDS #73-1665) and the presence of Si_2_ON_2_ (JCPDS #72-1307) and Si_3_N_4_ (JCPDS #71-0623) phases, respectively (Fig. 1a).^30,31^ As shown in previous reports, the polymer silicone resin poly(methyl phenyl silsesquioxane) converts to an amorphous SiOC phase at 1000 °C, which is further separated into SiC_4_, SiO_4_, and free carbon.^30,31^ When the pyrolysis temperature increases to 1400 °C, SiO volatilization is highly preferred along with CO gases from the SiOC matrix. The whole pyrolysis process occurs under the N_2_ atmosphere, and the incorporation of emanating SiO, CO, and N_2_ gases lead to supersaturation of its liquid phase and then forming the crystals of SiC, Si_2_ON_2_, and Si_3_N_4_, phase respectively, through solution precipitation *via* vapor–liquid–solid (VLS) mechanism.^30,31^ The presence of metal salt under these conditions strongly favors the formation of the corresponding metallic silicide, which is Ni_2_Si (JCPDS #48-1339) for Ni/PDC, Co_2_Si (JCPDS #89-4181) for Co/PDC, and Fe_3_Si (JCPDS #65-0994) for Fe/PDC, respectively. These metal salts in the presence of carbon species help in the reduction of Si–O bonds, and then the reduced Si atoms diffuse into the metallic atoms and form the corresponding metallic silicide.^36,37,42^Fig. 1b shows the Raman spectra of Ni/PDC, Co/PDC, and Fe/PDC samples, which contains the main characteristic disordered peak (D) at 1323 cm^−1^, and the graphitic peak (G) at 1593 cm^−1^, confirming the formation of free carbon in the PDC microstructure. In addition, the broad peaks at 2633 and 2891 cm^−1^, namely 2D and D + G peaks, endorse the signature of amorphous carbon in the matrix.^30,31^

**Fig. 2:**
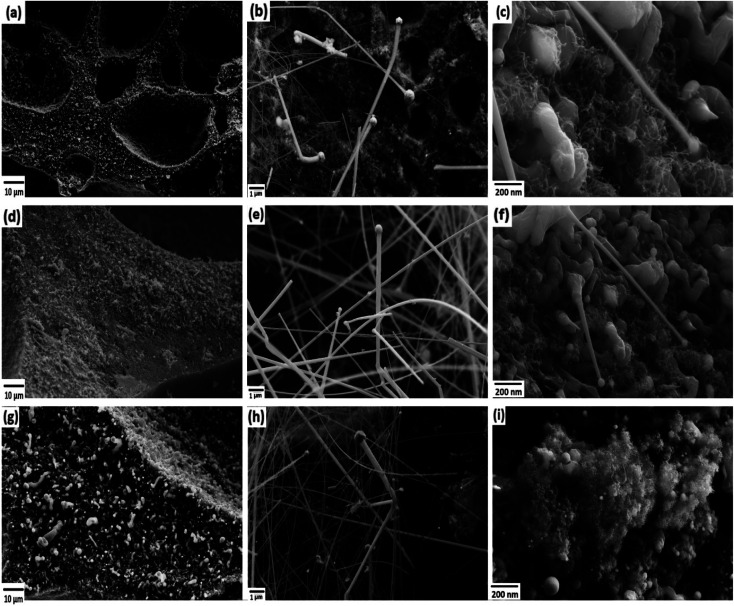
FESEM images of the ceramic samples (a–c) Ni/PDC, (d–f) Co/PDC, and (g–i) Fe/PDC.

**Fig. 3:**
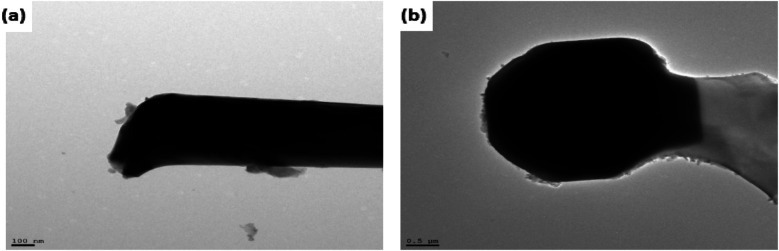
FETEM images of the (a) PDC and (b) Co/PDC samples.

**Fig. 4:**
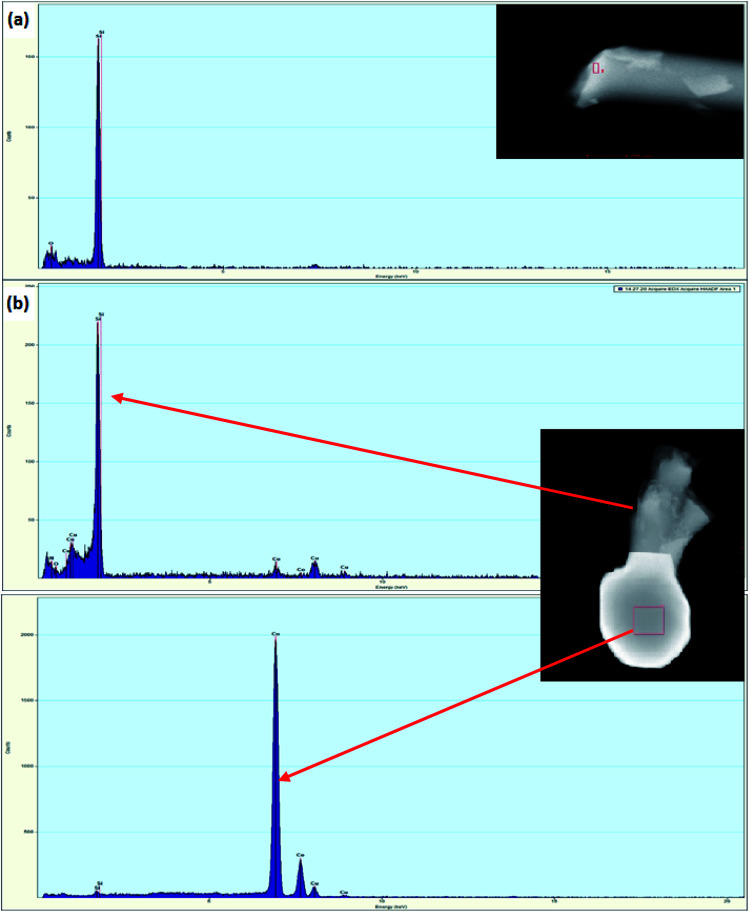
FETEM images/EDX analysis of (a) PDC and (b) Co/PDC (EDX spectra taken from selected area of top: nanowire and bottom: spherical tip).

The FESEM images of PDC samples are shown in Fig. 2. In a previous work, we demonstrated that transition metal nanoparticles initiate the formation of 1D-CNT within the porous PDCs when the pyrolysis temperature reaches 800–1000 °C.^37^ In this work, along with the cross-linking agent APTES, the foaming agent ADA was also used, which even influences the rheological properties of the preceramic polymer and helps the decomposition behavior. ADA creates a higher amount of porosity, which later allows the release of decomposition gases and supports the growth mechanism of 1D CNT/-PDC.^29^ The morphology of PDC samples pyrolyzed at 1400 °C under N_2_ atmosphere can be described as macrocellular PDC foam, which was completely decorated with large bundles of 1D ceramic nanowires with a length of 2–10 μm and a diameter of 50–400 nm (Fig. 2 and S1, ESI†). According to FETEM (Fig. 3 and 4) and FESEM (Fig. S2 and S3†) investigations, the EDX profiles taken at different spots of the nanowires show the presence of silicon, nitrogen, and oxygen, which confirms that the nanowires are made of Si_2_ON_2_ and Si_3_N_4_ phases. Along with ceramic nanowires, the metal-containing PDCs show the presence of spherical metal silicide droplets at the tips of ceramic nanowires (Fig. 4 and S2, S3†). As shown in these figures, the spherical droplets of Co/PDC samples are composed of both cobalt and silicon, which form a cobalt silicide (Co_2_Si). Likewise, the Ni/PDC samples are composed of both nickel and silicon that form Ni_2_Si, whilst no spherical droplets were noticed for metal free PDCs.^43^ Irrespective of various metal salts (Ni, Co, Fe, and Mn) added into the preceramic polymer, no substantial changes were observed in the shape and size of nanowires and spherical droplets. Moreover, the FESEM images of metal-containing PDC also show the *in situ* grown CNT with diameters of 10–60 nm and lengths of a few micrometres (Fig. 2c, f and i). The growth mechanism of ceramic nanowires and metallic silicide droplets at the tips follow the VLS mechanism, which is already explained in the literature.^30,31^ In this metal salt (Ni, Co, Fe, and Mn) containing PDC matrix, during the pyrolysis (600–1000 °C) the metal salt was reduced to metallic nanoparticles through carbothermal reduction. Then, these metal nanoparticles help the formation of 1D CNT through the catalyst-assisted pyrolysis in which decomposition, diffusion, and precipitation of hydrocarbon occur as the 1D CNT on metal catalysts is formed. When the pyrolysis temperature increases to 1000 °C, the metal nanoparticles start to diffuse into silicon-containing matrix and form metallic silicide that is in a pseudo fluid state at temperatures above 1200 °C. At this juncture, the liquid metallic silicide catalyst collects the decomposition gases such as SiO and CO evolved from PDC ceramics and atmospheric N_2_ gas, which stimulates the formation of the PDC nanowires.^30,31^ The N_2_ adsorption–desorption isotherms of PDC samples pyrolyzed at 1400 °C under N_2_ atmosphere show a typical type II isotherm (Fig. S4†). Among them, Ni/PDC shows the highest BET surface area of 34 m^2^ g^−1^ followed by Co/PDC and Fe/PDC, respectively.

**Fig. 5:**
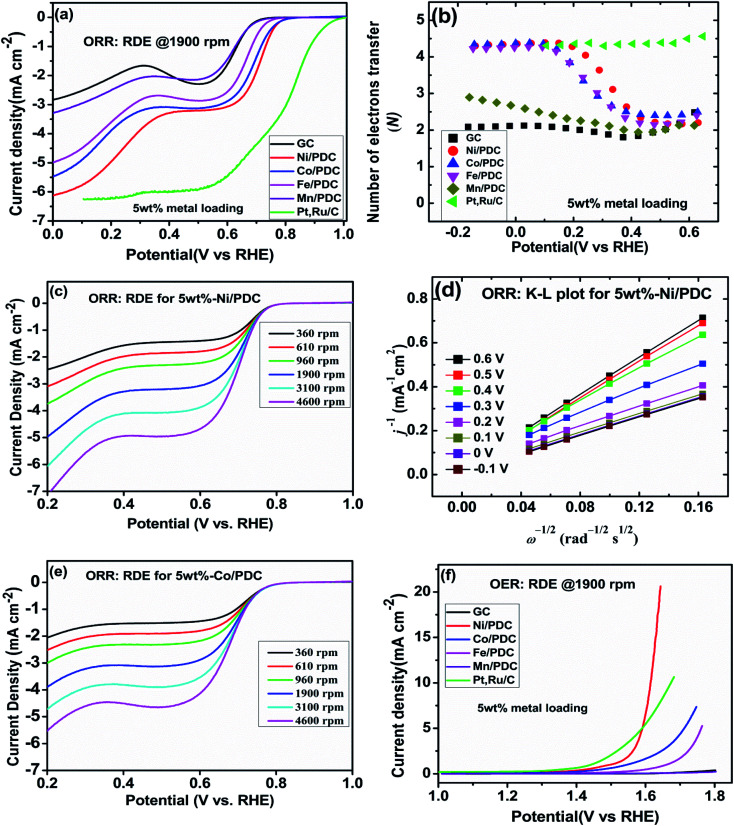
(a) RDE polarization curves for O_2_ reduction on different electrodes in O_2_-saturated 0.1 M KOH (*ω* = 1900 rpm, *ν* = 10 mV s^−1^), (b) corresponding potential dependence of the number of electrons transferred per O_2_ molecule calculated using the K–L equation, (c and e) RDE polarization curves for O_2_ reduction of the Ni/PDC and Co/PDC catalyst in O_2_-saturated 0.1 M KOH (*ω* = 360; 610; 960; 1900; 3100; 4600 rpm, *ν* = 10 mV s^−1^), (d) corresponding K–L plots for Ni/PDC, and (f) RDE polarization curves for O_2_ evolution on different catalyst materials in O_2_-saturated 0.1 M KOH (*ω* = 1900 rpm, *ν* = 10 mV s^−1^).

**Fig. 6:**
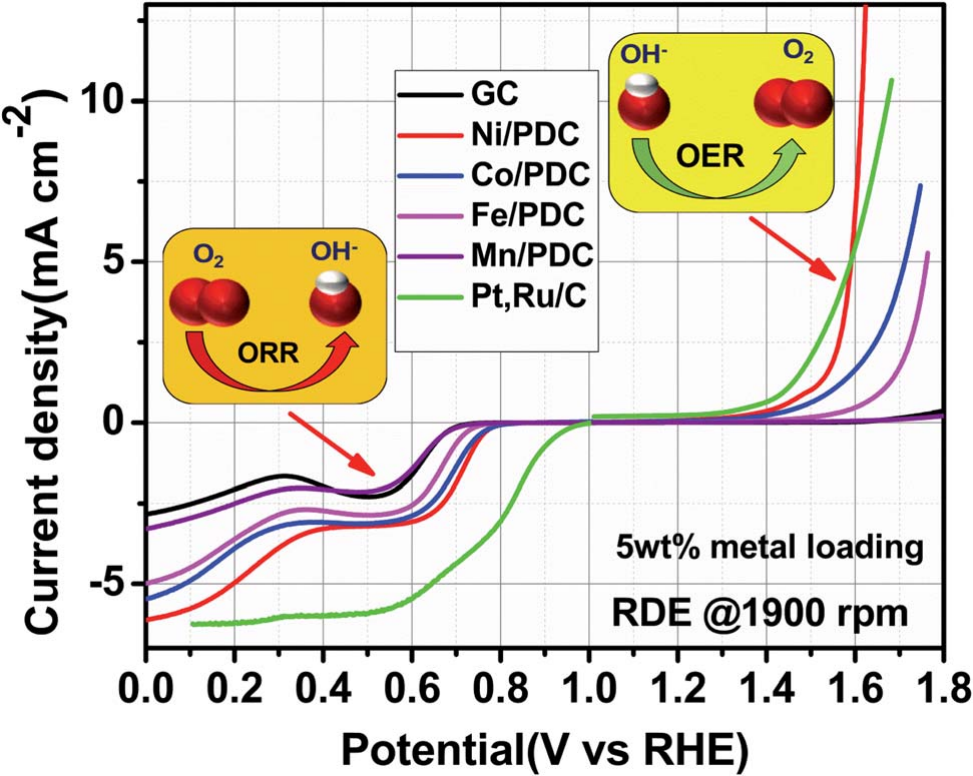
RDE polarization curves for oxygen reduction and oxygen evolution on GC electrodes coated with different catalyst materials in O_2_-saturated 0.1 M KOH (*ω* = 1900 rpm, *ν* = 10 mV s^−1^).

**Fig. 7:**
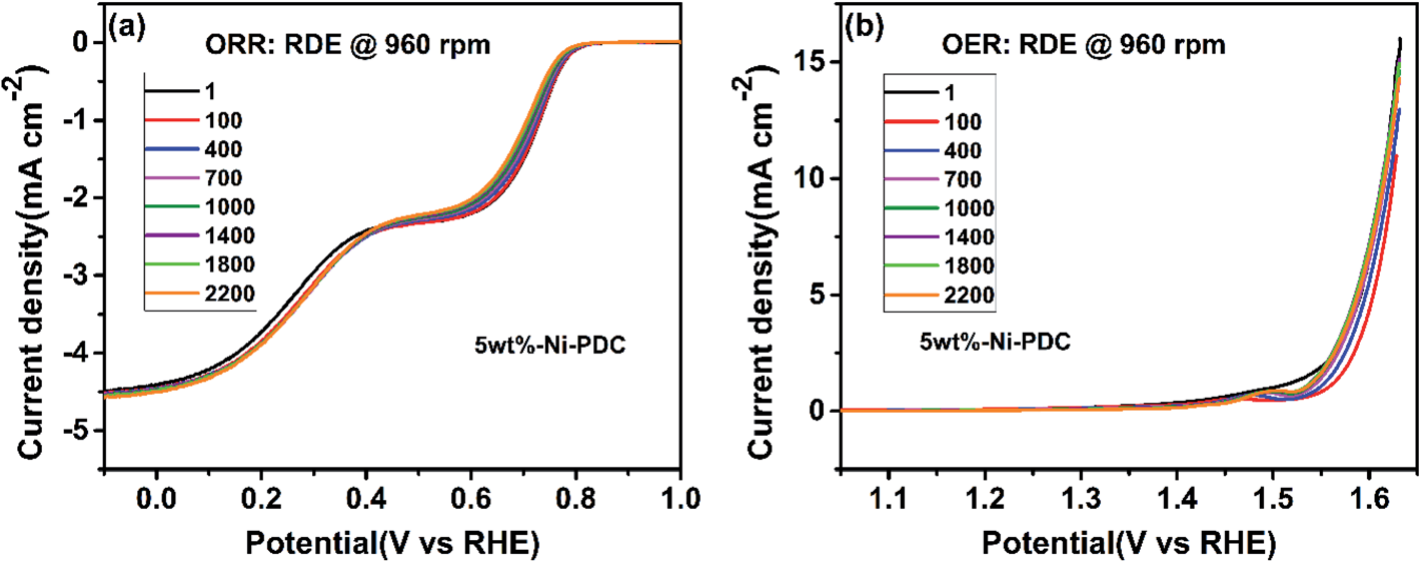
Stability testing of: (a) ORR and (b) OER on the 5 wt%-Ni-PDC-coated GC electrode in (a) O_2_- and (b) Ar-saturated 0.1 M KOH. The number of the RDE voltammetry curve (*ω* = 960 rpm, *ν* = 10 mV s^−1^) shows how many (a) CV (*ν* = 100 mV s^−1^) and (b) RDE voltammetry (*ν* = 100 mV s^−1^, *ω* = 960 rpm) potential cycles were performed prior to recording the shown curve.

The effect of metallic silicide droplets at the tips of 1D PDC nanowires towards the electrochemical bifunctional activity, *i.e.*, the half-cell studies were performed in a standard three-electrode system.^44^ In Fig. S5 and S6,† the cyclic voltammograms recorded in the non-faradaic region for 5 wt% and 10 wt% metal, respectively, containing PDC catalyst coated electrodes are shown. The expected linear dependence between cathodic current at 0.915 V and the scan rate is shown in Fig. S5 and S6.† Also, the *C*_dl_ values were determined and the highest values were obtained in the case of Ni containing PDC catalysts. The higher *C*_dl_ value indicates that the catalyst can host more electroactive sites compared to the materials that have lower *C*_dl_ values.^45^ The ORR polarization curves are shown after subtraction of the background current measured in N_2_-saturated electrolyte solution. The highest ORR onset potential and lowest OER overpotential together with improved activity were obtained in preliminary tests in the case of 5 wt% metal (Ni, Co, Fe, and Mn) loading compared with higher metal loading (10 wt%) and therefore, 5 wt% metal loading was used as the optimum metal loading of PDC to obtain a good electrocatalytic activity (Fig. 5, 6 and S7†). Fig. 5a depicts the electrocatalytic activity of the prepared catalyst along with Pt, Ru/C for O_2_ reduction recorded in O_2_-saturated 0.1 M KOH at 1900 rpm. It is striking that both Ni/PDC and Co/PDC catalysts display efficient ORR performance in terms of both onset and half-wave potential. The Ni/PDC shows a good ORR activity with approximately 50 mV higher half-wave potential (*E*_1/2_) compared to that of Co/PDC, but was not superior to Pt, Ru/C catalyst. The corresponding potential dependence of the number of electrons transferred per O_2_ molecule was determined between −0.1 and 0.6 V *vs.* RHE for different electrodes calculated from the Koutecky–Levich (K–L) eqn (2)^46^ and the results are shown in Fig. 5b.2
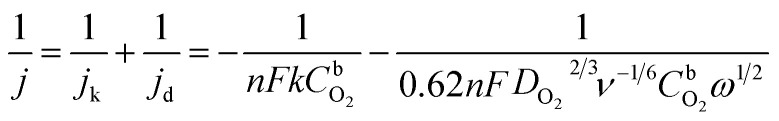
where *j* is the overall measured current density, *j*_k_ and *j*_d_ are the kinetic and diffusion-limited current densities, respectively, *k* is the electrochemical rate constant for oxygen reduction, *F* is the Faraday constant (96 485 C mol^−1^), *ω* is the rotation rate, 
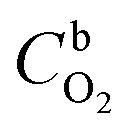
 is the O_2_ concentration in the bulk (1.2 × 10^−6^ mol cm^−3^),^47^*D*_O2_ is the O_2_ diffusion coefficient (1.9 × 10^−5^ cm^2^ s^−1^),^47^ and *v* is the kinematic viscosity of the electrolyte solution (0.01 cm^2^ s^−1^).^48^Fig. 5b shows that the ORR proceeds mainly *via* the 2-electron pathway at lower overpotentials (0.6 > *E* > 0.3 V) and *via* the 4-electron pathway at higher overpotentials (0.3 > *E* > −0.1 V).^49^ Similar electrochemical ORR behavior has been observed with pristine and transition metal (Co and Ni) doped SiOC ceramic catalyst.^50^Fig. 5c–e shows the oxygen reduction performance of Ni/PDC and Co/PDC catalyst at different rotation rates. As the rotation rate increases, the higher the mass transport rate of oxygen to the catalyst-coated GC electrode, and therefore, the ORR current density also increases.^51^ The corresponding K–L plots for Ni/PDC do not coincide, with the extrapolated lines intersecting the *j*^−1^ axis in the positive, indicating that the ORR occurs under the mixed kinetic-diffusion control (Fig. 5d).^50^ In the case of OER, the Ni/PDC sample outperforms the standard Pt, Ru/C catalyst and is significantly more active than the other transition metal doped catalysts. Ni/PDC exhibits the lowest potential of 1.61 V *vs.* RHE compared with Pt, Ru/C (1.67 V *vs.* RHE) when the current density reaches 10 mA cm^−2^ (Fig. 5f). The other transition metal (Co, Fe, and Mn) doped catalysts do not reach 10 mA cm^−2^ current density within the measured region (1.8 V > *E*), which confirms that Ni/PDC is a more suitable OER catalyst.^52^ To evaluate the oxygen electrode activity of PDC catalyst, the bifunctional ability of the catalyst is evaluated by calculating Δ*E* value by the difference in the potential from OER at 10 mA cm^−2^ and potential from ORR at −3 mA cm^−2^ (Fig. 6 and Table 1).^53,54^ The Δ*E* of 0.99 V for 5 wt% Ni/PDC shows a slightly lower performance compared to the expensive state-of-the-art catalyst Pt, Ru/C with 0.87 V, concluding that Ni/PDC acts as a cheap and efficient bifunctional electrocatalyst for the Zn–air battery. A comparison of Δ*E* values demonstrate that the electrocatalytically active Ni_2_Si droplets at the tips of ceramic nanowires and the growth of 1D CNT/-PDC promote better ORR and OER kinetics. Moreover, the multivalent nickel ions present in the Ni/PDC catalyst improves the reaction kinetics as Ni^2+^ ions promote the adsorption of O_2_ and the reduction process and Ni^3+^ ions enhance the OER kinetics. Also, the ORR and OER stability of 5 wt%-Ni/PDC catalyst was evaluated using long-term potential cycling in 0.1 M KOH solution (Fig. 7).

**Table 1:** The potential at ORR current density of −3 mA cm^−2^ (*E*_ORR_), potential at OER current density of 10 mA cm^−2^ (*E*_OER_) and the ORR/OER reversibility (Δ*E* = *E*_OER_ − *E*_ORR_) values for different catalyst material-coated GC electrodes obtained from Fig. 6 and S7

Electrode	*E*_ORR_ (V)	*E*_OER_ (V)	Δ*E* (V)
5 wt%-Ni/PDC	0.62	1.61	0.99
10 wt%-Ni/PDC	0.58	1.64	1.06
Pt, Ru/C	0.80	1.67	0.87

The catalyst stability was studied by applying 2200 CV (100 mV s^−1^) and RDE voltammetry (100 mV s^−1^, 960 rpm) cycles for ORR and OER experiments, respectively. In the case of both studies, the RDE polarization curve was recorded using 960 rpm and 10 mV s^−1^ after every 100 cycles. One can see that during 2200 cycles, the ORR and OER onset potentials have shifted to slightly more negative and positive potentials, respectively. Also, the *C*_dl_ value was determined after the ORR and OER stability testing, resulting in 98% and 73% of the initial *C*_dl_ value, respectively. Also, the STEM experiments were performed with the 5 wt%-Ni/PDC catalyst after the stability testing. The high-angle annular dark field (HAADF) images along with the EDX mapping (Fig. S8†) show the spherical Ni silicide droplets with different sizes being still present after the stability test as in the original Ni/PDC material (Fig. 2a–c and S2†). In general, the 5 wt%-Ni/PDC bifunctional catalyst shows good stability in 0.1 M KOH electrolyte solution.

EDX mapping showed that the iron is mostly inside the nanotubes or covered with the graphitic layers for both catalysts (Fig. 2a). The half-cell studies affirm that the prepared catalysts exhibit good bifunctional activity and thus become an appropriate material to be employed as an air electrode in the Zn–air battery construction.

**Fig. 8:**
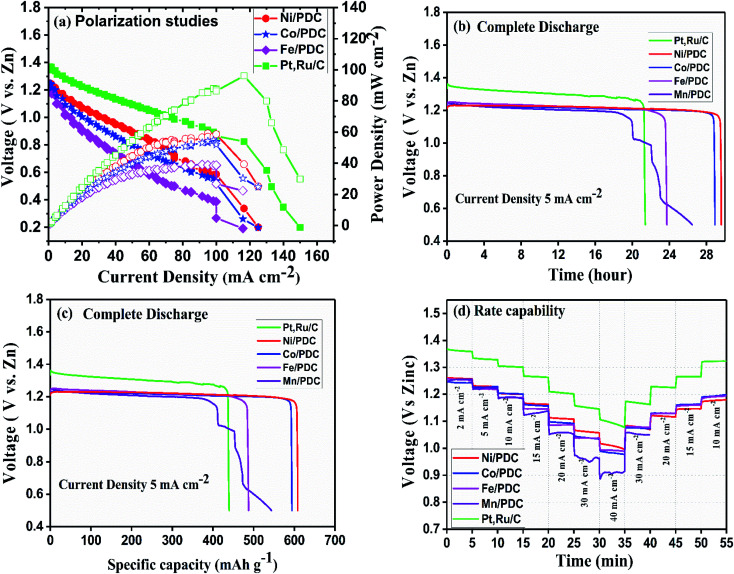
Primary Zn–air battery performance of the ceramic cathode catalyst. (a) Polarization and power density curves, (b) complete discharge as a function of time, (c) complete discharge as a function of specific capacity, and (d) rate capability studies.

The ability of the prepared ceramic catalysts to perform in a Zn–air battery environment was determined in a primary and mechanically rechargeable configuration. The ceramic catalyst was brush coated upon the GDL layer using Nafion ionomer. The battery was built by placing a microporous polyolefin separator (dipped in 6 M KOH) in between a 1 mm thick Zn sheet and a catalyst coated GDL. This construction helps in creating a three-phase boundary between the electrolyte, catalyst, and reactant air. The discharge polarization graph Fig. 8a clearly shows that Ni/PDC has the highest peak power density of 59 mW cm^−2^ followed by Co/PDC with a power density of 55 mW cm^−2^ and Fe/PDC with a power density of 39 mW cm^−2^, respectively. The peak power density of ceramic samples was lower compared to the state-of-the-art catalyst Pt, Ru/C that has a peak power density of 96 mW cm^−2^. The galvanostatic discharge curves (Fig. 8b and c) of all prepared ceramic catalysts and Pt, Ru/C were recorded to understand the role of the air electrode in promoting the efficiency in primary battery configuration. The open-circuit voltages were observed to be 1.37 V > 1.24 V > 1.22 V for Pt, Ru/C > Ni/PDC > Co/PDC samples, which are lower than the theoretical zinc–air battery potential of 1.65 V.^11^ The studies showed that Ni/PDC and Co/PDC samples exhibited a stable discharge voltage plateau up to 29 h at a potential of 1.24 V while with Fe/PDC and Mn/PDC, the battery stopped working after less than 23 h at the current density of 5 mA cm^−2^. It can also be noted that though the Pt, Ru/C discharge plateau potential is 130 mV higher than that of Ni/PDC yet, it has a short discharge capacity up to 8 h lesser than that of Ni/PDC. Further, from Fig. 8c, it is observed that the specific capacity of Ni/PDC is 608 mA h g_Zn_^−1^, which is much higher than the specific capacity of Co/PDC 593 mA h g_Zn_^−1^, Fe/PDC 485 mA h g_Zn_^−1^, and Pt, Ru/C 437 mA h g_Zn_^−1^, respectively. Fig. 8d shows the C-rate capability of the ceramic samples. It is obvious that the ceramic catalyst maintains a similar discharge potential window, both for step-down/step-up rate discharge processes and at a high discharge rate of 40 mA cm^−2^, which significantly suggests that the ceramic sample possesses better stability. These results strongly conclude that Ni/PDC, Co/PDC, and Fe/PDC perform better in real-time primary Zn–air battery, which is due to the presence of active metallic silicide nanoparticles on the tips of the ceramic nanowires and *in situ* grown CNTs that possess good electrical conductivity and feasible ORR kinetics.^37^

**Fig. 9:**
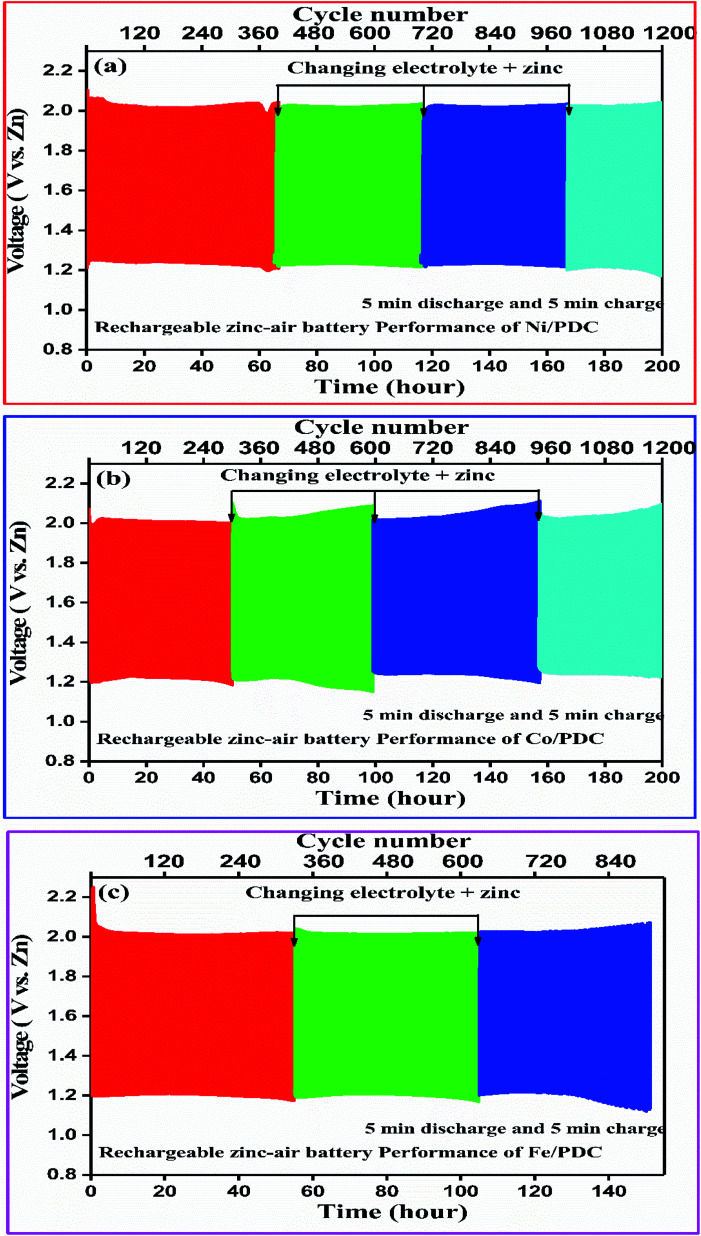
Mechanically rechargeable Zn–air battery performance of the (a) Ni/PDC, (b) Co/PDC and (c) Fe/PDC catalyst at a current density of 5 mA cm^−2^.

Fig. 9 shows the 5 minute galvanostatic charge–discharge cycle of Ni/PDC, Co/PDC, and Fe/PDC samples. The charge–discharge cycle of Ni/PDC at 5 mA cm^−2^, which clearly shows that the initial discharge/charge potential was 1.24 V/2.03 V, while the calculated round-trip efficiency of about 61% is stable until the battery operation up to 65 h (Fig. 9a). In the case of Co/PDC shown in Fig. 9b, the charging and discharging voltages are 2.02 V and 1.20, and the battery works up to 49 h without increasing the overpotential. Even Fe/PDC based Zn–air battery performs well up to 54 h (Fig. 9c). The commercial Pt, Ru/C catalyst (Fig. S9†) was studied for reference purposes where the battery ceased to perform beyond 30 h. Initially, the charging and discharging voltages were 1.9 V and 1.33 V, which eventually increased up to 1.99 V and 1.21 V after 30 h, reflecting the instability of Pt, Ru/C catalyst. During long-term cycling, the formation of ZnO on the surface of the anode causes the Zn–air battery to seize operation.^55,56^ In order to understand the stability of the prepared catalysts, Zn–air battery measurements were carried out after the replacement of zinc and electrolyte when the battery ceased to perform. In the mechanical rechargeable Zn–air battery using Ni/PDC and Co/PDC cathode catalysts, 1200 stable galvanostatic charge–discharge cycles were performed at a current density of 5 mA cm^−2^ by replacing the zinc and electrolyte four times (Fig. 9a and b). Similar studies have utilized Fe/PDC (Fig. 9c) as a cathode catalyst where it performed 900 stable charge and discharge cycles by replacing the zinc and the electrolyte three times. As a significant result, we have found a mechanically rechargeable Zn–air battery configuration by replacing fresh zinc anodes and electrolyte, whereby similar battery performance can be reproduced without any catalyst degradation. These results conclude that Ni/PDC, Co/PDC, and Fe/PDC act as suitable bifunctional catalysts with the ability to exhibit superior Zn–air battery performance. The superior catalyst activity in this work is possibly due to the well-constructed metallic silicide droplets on the tips of the 1D ceramic nanowires, which guarantees both active sites for reaction kinetics and conduction path for electrons. The incorporation of metal particles within the PDC ceramics enables the formation of 1D PDC/-CNT that enhances chemical, electrical, and thermal stability. In addition, these metallic silicide droplets are more active electrocatalytic sites for both ORR/-OER reaction kinetics and are formed on the tips of 1D PDC, facilitating the effective utilization of the catalyst. Thus, this unique architecture dramatically enhances the Zn–air battery in terms of both durability and performance.

## Conclusion

For the first time, new metallic nickel/cobalt/iron silicide droplets on the tips of the PDC nanowires have been successfully prepared and utilized as a cathode catalyst for Zn–air batteries. The Ni/PDC and Co/PDC cathode catalyst deliver a peak power density of ∼55–59 mW cm^−2^, exhibit a stable discharge voltage plateau up to 29 h at a potential 1.24 V, and show 1200 stable mechanically rechargeable cycles at a current density of 5 mA cm^−2^. The Fe/PDC also exhibits 900 stable mechanically rechargeable cycles projecting it to be a good electrocatalyst for Zn–air batteries. The good bifunctional ORR/OER activity of the PDC-based catalyst is due to the presence of electrocatalytically active intermetallic silicide nanoparticles on the tips of the 1D ceramic nanowires. These observations and results strongly affirm that Ni/PDC, Co/PDC, and Fe/PDC are potential candidates to be employed as cheap and efficient electrocatalysts for real-time Zn–air battery configurations.

## Conflicts of interest

The authors declare no conflict of interest.

## Supplementary Material

RA-011-D1RA05688C-s001
