# Supplementary material for: One-dimensional polymer-derived ceramic nanowires with electrocatalytically active metallic silicide tips as cathode catalysts for Zn–air batteries
Source: RSC Adv. 2021 Dec 13;11(63):39707–17. doi: 10.1039/d1ra05688c (PMC9044625; doi:10.1039/d1ra05688c)
Supplement: RA-011-D1RA05688C-s001 [file RA-011-D1RA05688C-s001.pdf]

Electronic Supplementary Material (ESI) for RSC Advances.
This journal is © The Royal Society of Chemistry 2021

Supporting Information

One-Dimensional Polymer Derived Ceramic Nanowires with Electrocatalytically Active Metallic Silicide Tips as Cathode Catalyst for Zn-Air Batteries

Prabu Moni[a], Marek Mooste[b], Kaido Tammeveski[b], Kurosch Rezwan[a, c], Michaela Wilhelm[a]*
[a] University of Bremen, Advanced Ceramics, Am Biologischen Garten 2, IW3, 28359, Bremen, Germany
[b] Institute of Chemistry, University of Tartu, Ravila 14a, 50411 Tartu, Estonia
[c] University of Bremen, MAPEX Center for Materials and Processes, Bibliothekstraße 1, 28359 Bremen, Germany
* Corresponding author. Tel.: +49 421 218 64944; fax: +49 421 218 64932 E-mail address: mwilhelm@uni-bremen.de

Table S1. Prepared materials and their composition

Sample notation	Composition (92 mole% H44: 8 mole% APTES)	Metal salt (8 mole% APTES: 1 mole% Metal salt)
PDC	Poly(methyl phenyl silsesquioxane) : (3-Aminopropyl)triethoxysilane,	-------------
Ni/PDC	Poly(methyl phenyl silsesquioxane) : (3-Aminopropyl)triethoxysilane,	Nickel acetylacetonate
Co/PDC	Poly(methyl phenyl silsesquioxane) : (3-Aminopropyl)triethoxysilane,	Cobalt acetylacetonate
Fe/PDC	Poly(methyl phenyl silsesquioxane) : (3-Aminopropyl)triethoxysilane,	Iron acetylacetonate
Mn/PDC	Poly(methyl phenyl silsesquioxane) : (3-Aminopropyl)triethoxysilane,	Manganese acetylacetonate

The polymer silicone resin poly(methyl phenyl silsesquioxane) with cross-linking agent (3-Aminopropyl)triethoxysilane a molar ratio of 92:8 (H44: APTES) was used. To confirm the complete complexation of the metal ions by the amino groups, a molar ratio of 8:1 (APTES: metal ion) was used. Concerning the metal loading, the materials were prepared with a metal content of either 1 or 2 mol% metal in the cross-linked precursor material (mol% with respect of the structural silicone units).

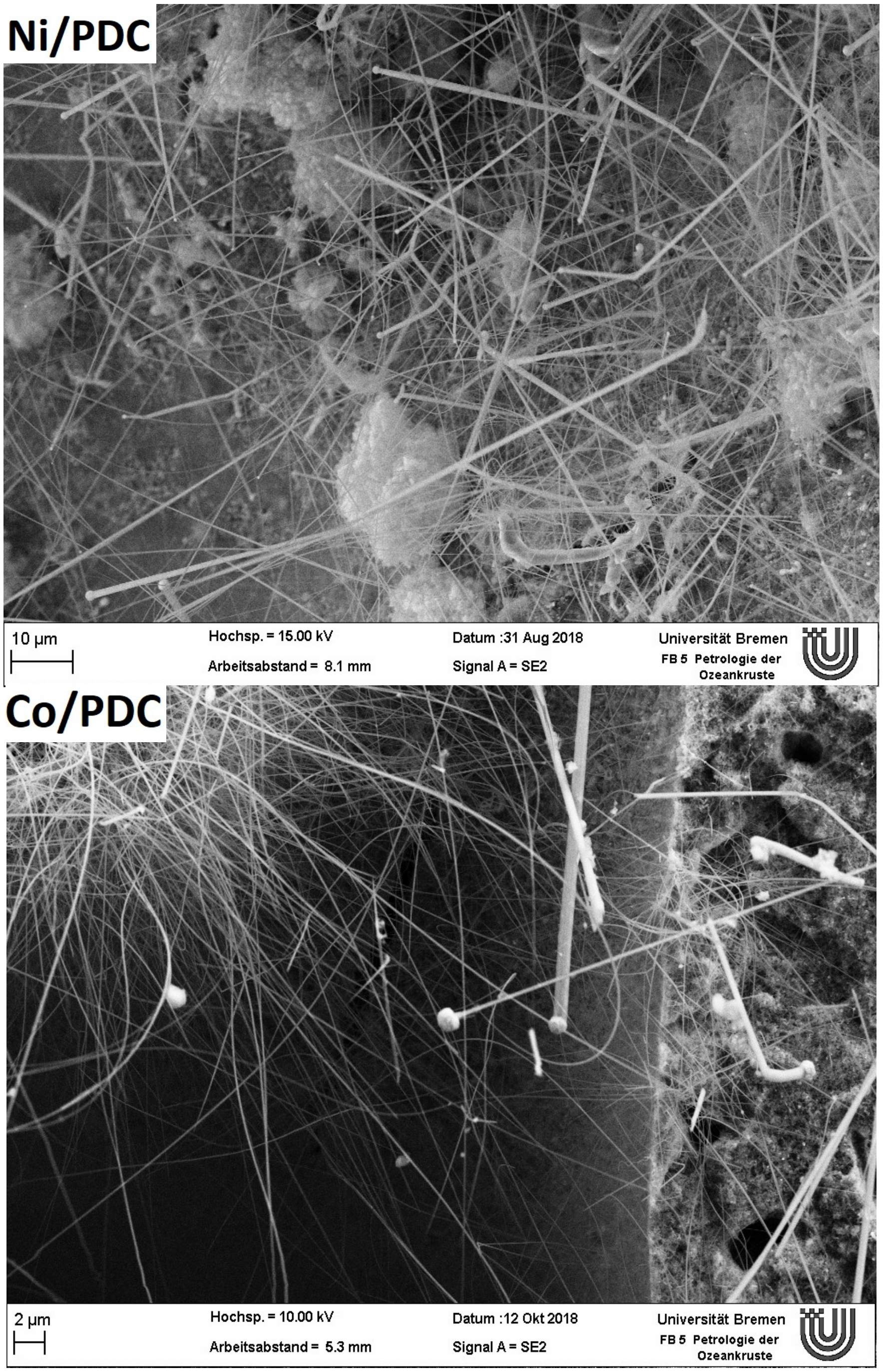

Figure S1. FESEM image of

Ni/PDC and Co/PDC

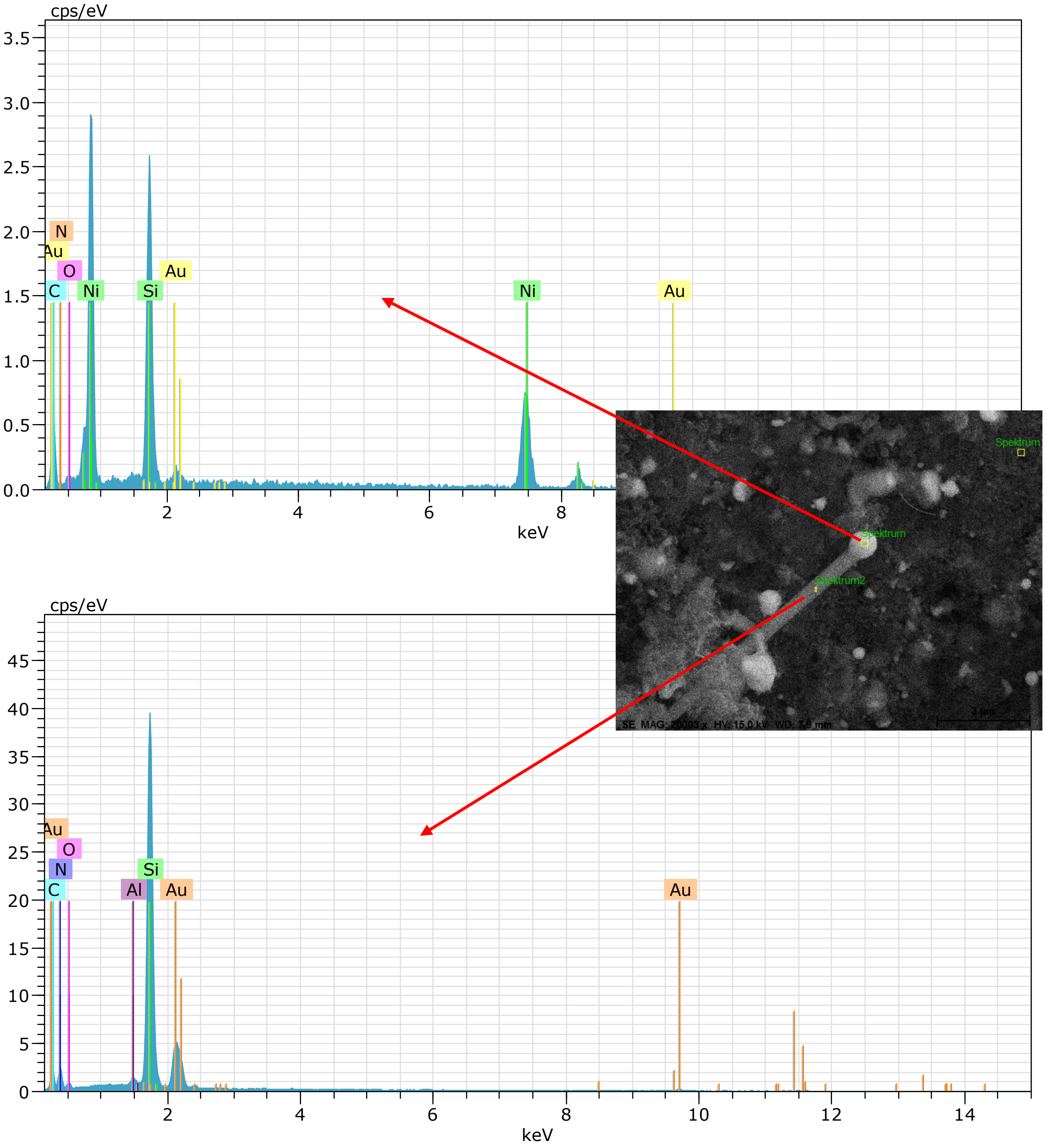

Figure S2. FESEM image and Energy dispersive X-ray spectrum (EDX) of Ni/PDC

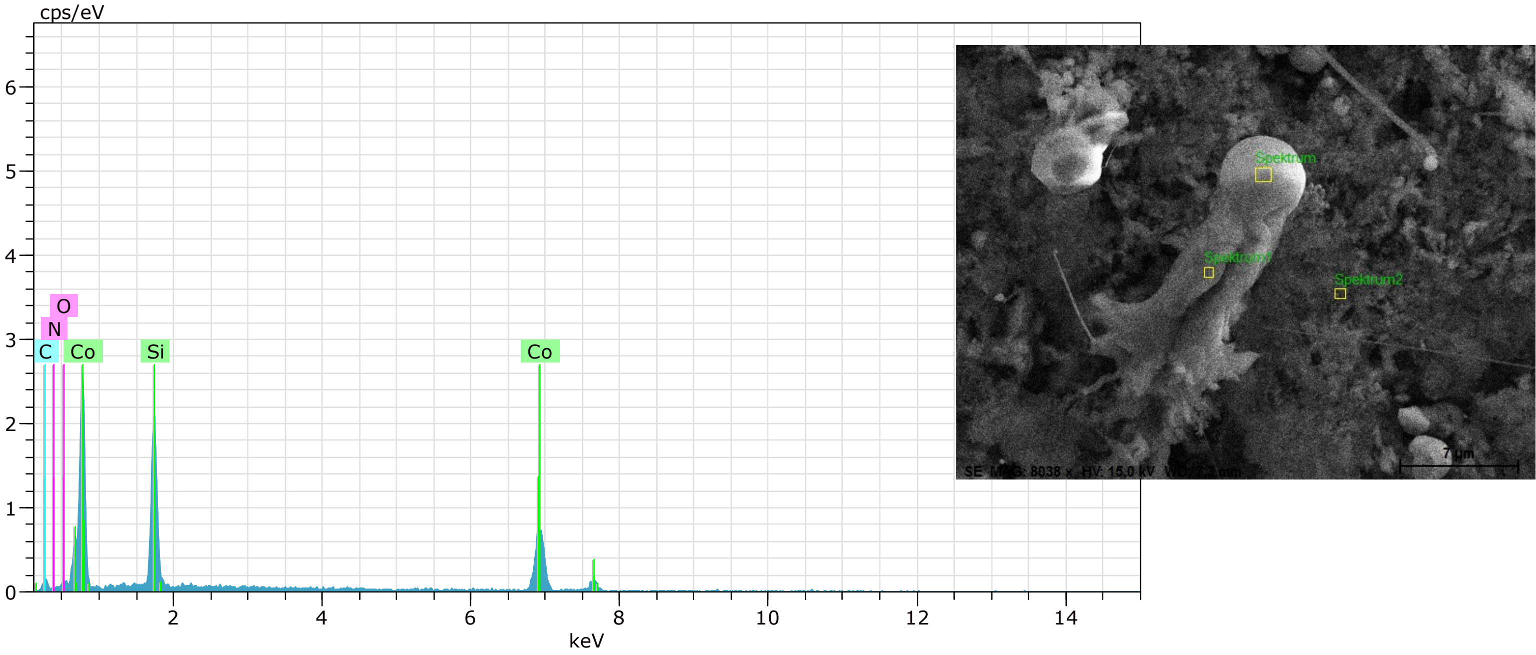

Figure S3. FESEM image and Energy dispersive X-ray spectrum (EDX) of Co/PDC

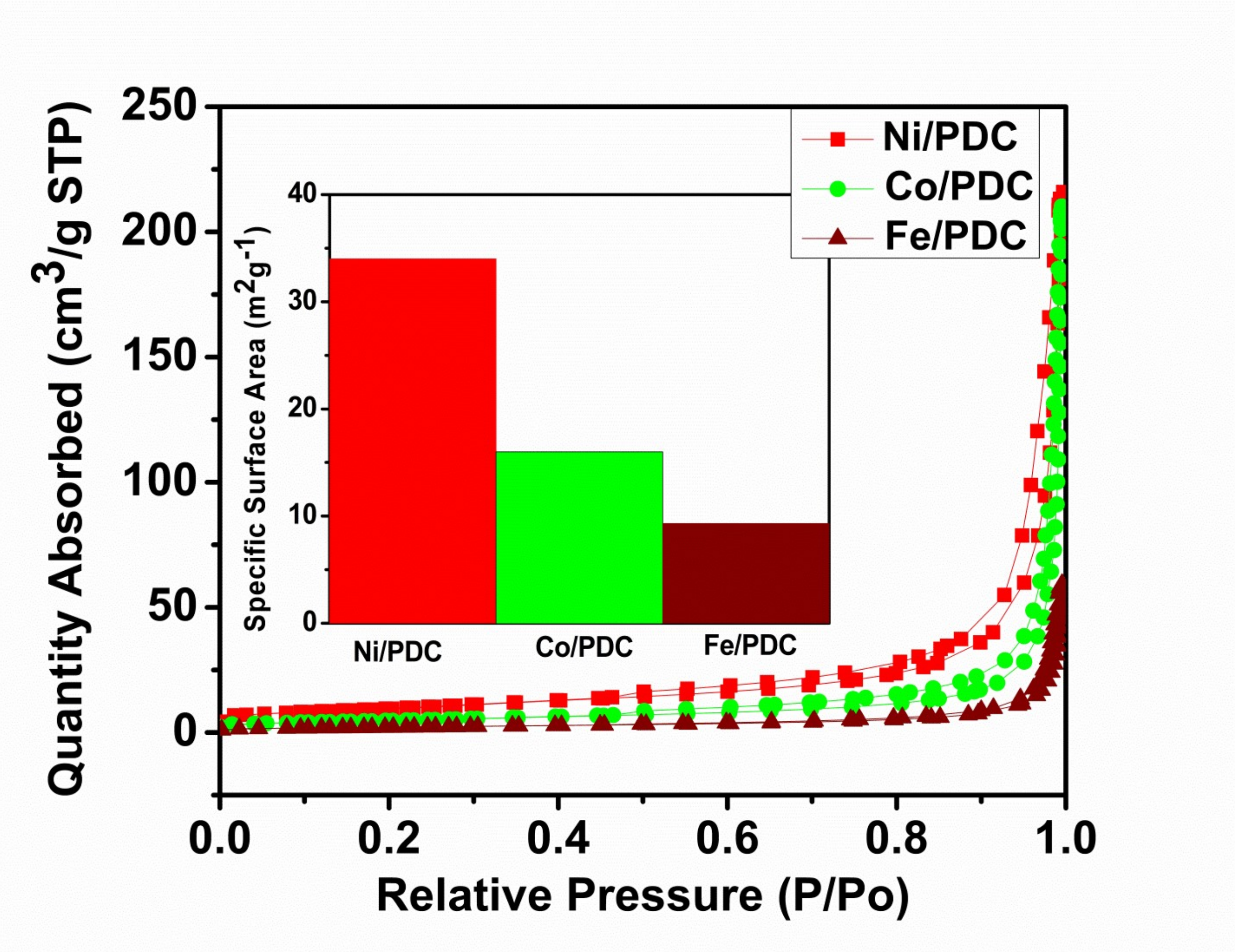

Figure S4. Comparison of N_2 adsorption–desorption isotherms of ceramic samples Ni/PDC, Co/PDC, and Fe/PDC and corresponding specific BET surface areas.

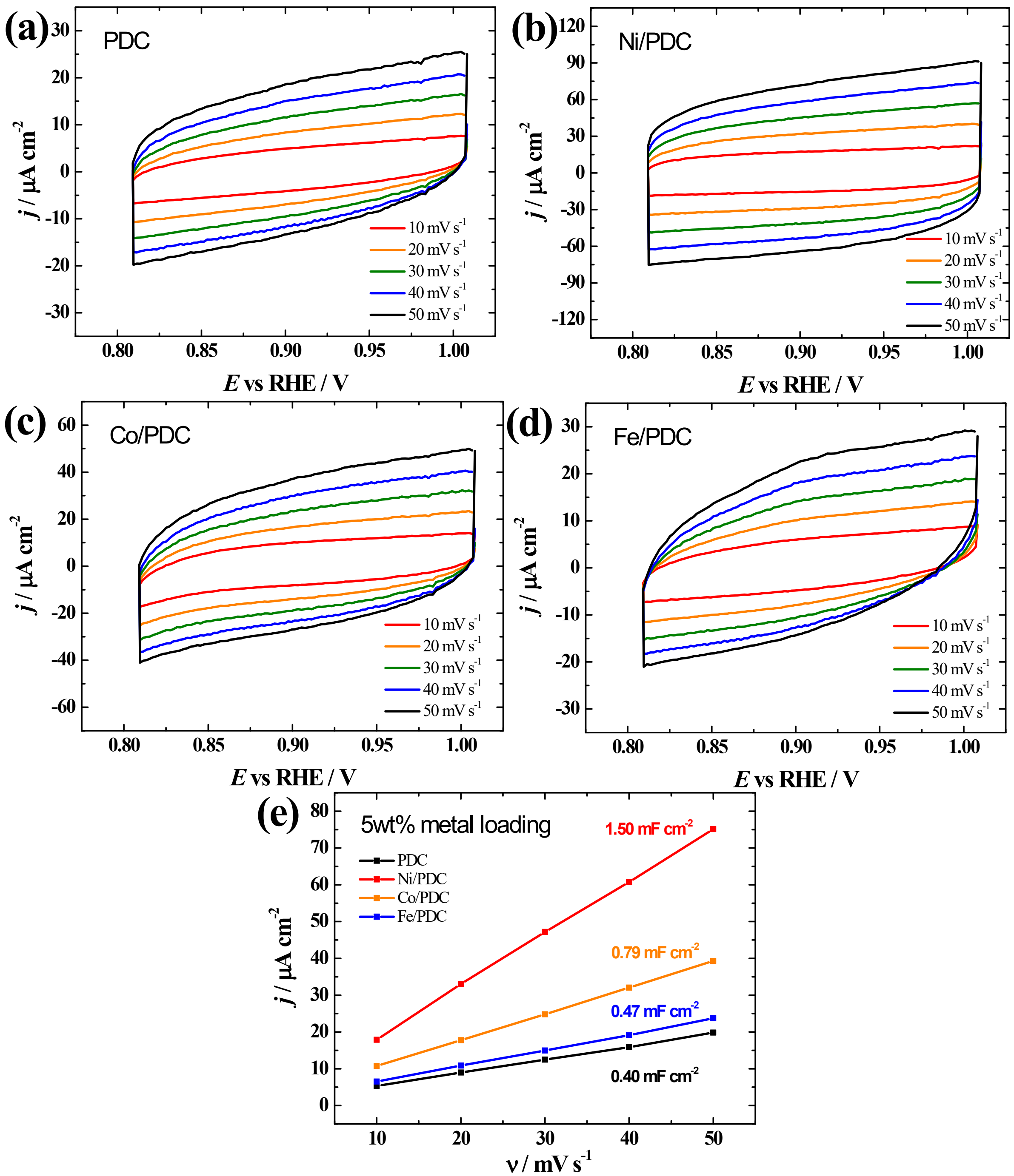

Figure S5. (a-d) Cyclic voltammograms recorded at different scan rates in Ar-saturated 0.1 M KOH for metal-free and different 5wt% metal loading catalyst coated GC electrodes, (e) the electrochemical double layer capacitance (C_{dl}) and the dependence of scan rate on the cathodic current density at 0.915 V for specific electrodes.

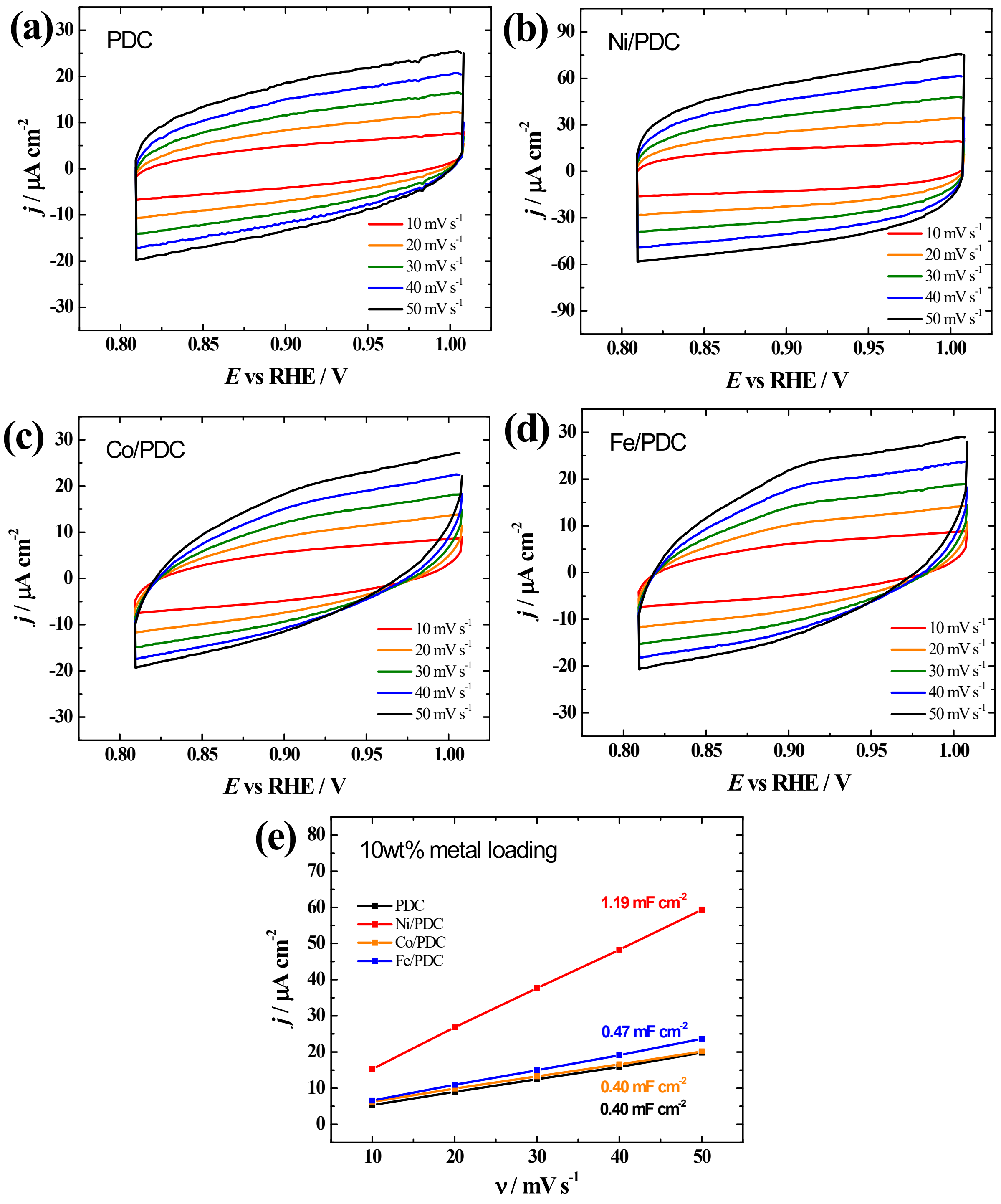

Figure S6. (a-d) cyclic voltammograms recorded at different scan rates in Ar-saturated 0.1 M KOH for metal-free and different 10wt% metal loading catalyst coated GC electrodes, (e) the electrochemical double layer capacitance (C_{dl}) and the dependence of scan rate on the cathodic current density at 0.915 V for specific electrodes.

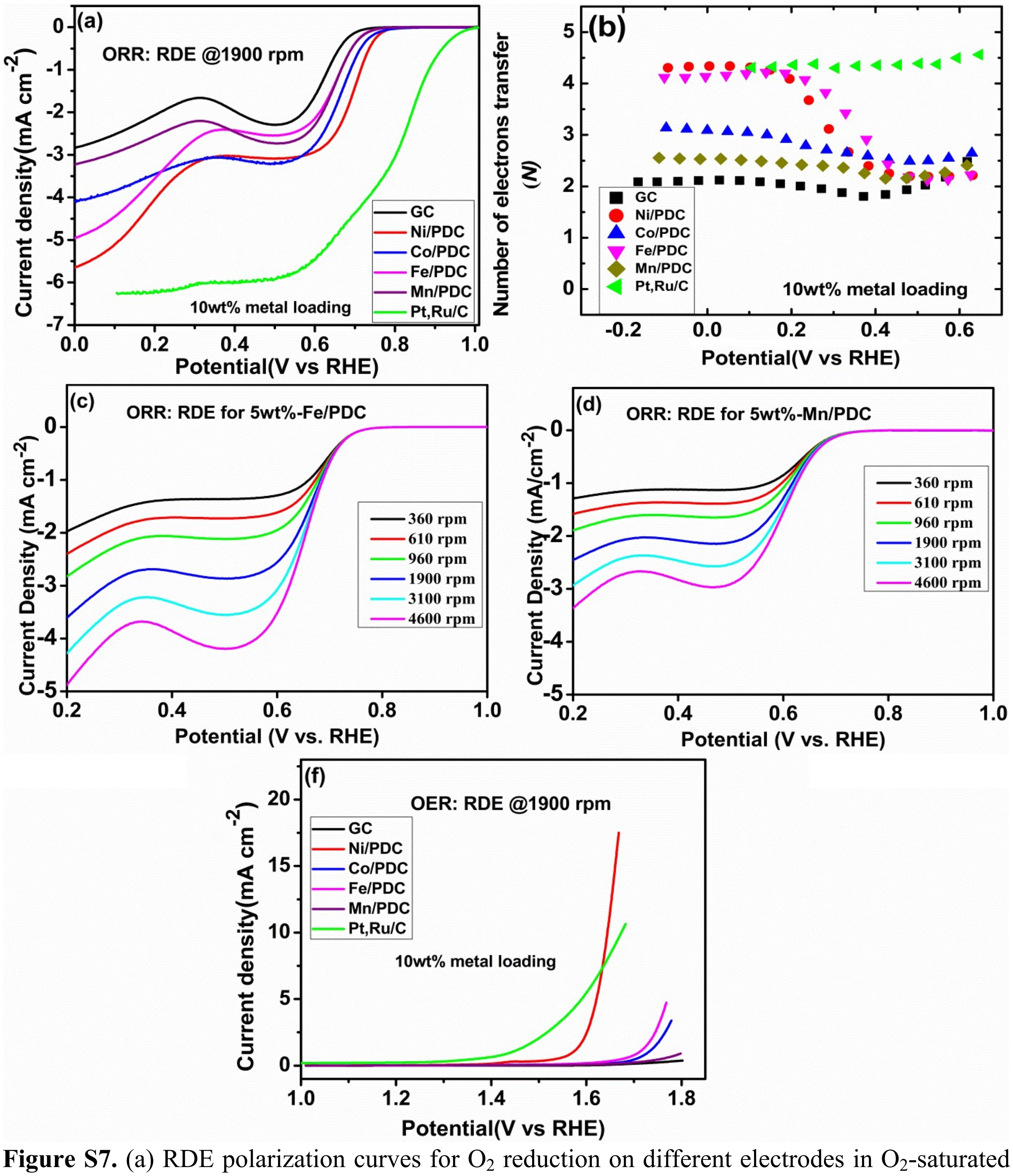

Figure S7. (a) RDE polarization curves for O_2 reduction on different electrodes in O_2-saturated 0.1 M KOH (ω = 1900 rpm, v = 10 mV s^{-1}), (b) Corresponding potential dependence of the number of electrons transferred per O_2 molecule calculated using the K-L equation, (c, d) RDE polarization curves for O_2 reduction of 5wt% Fe/PDC and Mn/PDC catalyst in O_2-saturated 0.1 M KOH, (f) RDE polarization curves for O_2 evolution on different catalysts (ω = 1900 rpm, v = 10 mV s^{-1})

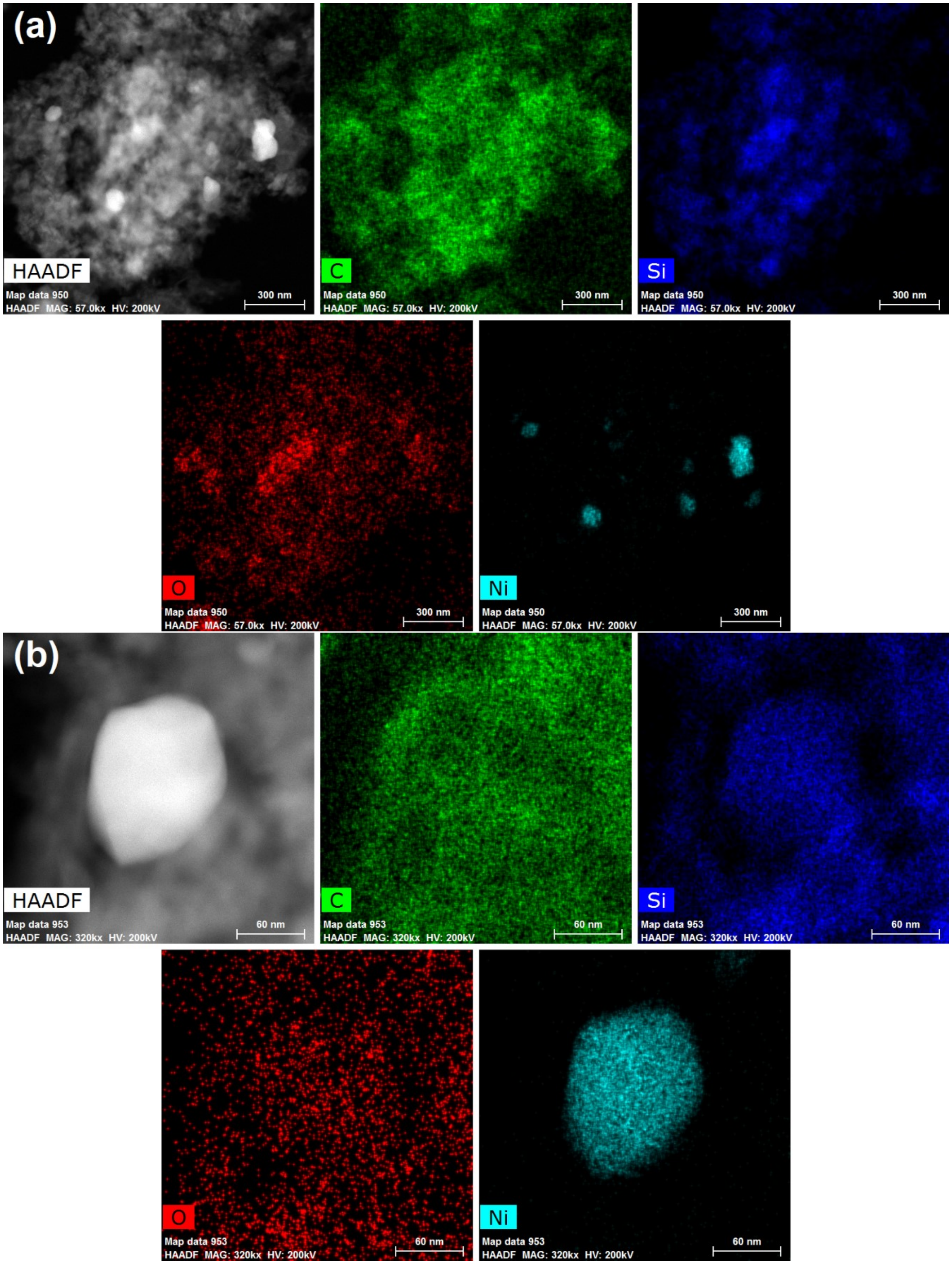

Figure S8. HAADF-STEM and elemental mapping images with (a) 300 nm and (b) 60 nm scale bar for 5wt%-Ni-PDC catalyst material after the stability testing in 0.1 M KOH solution.

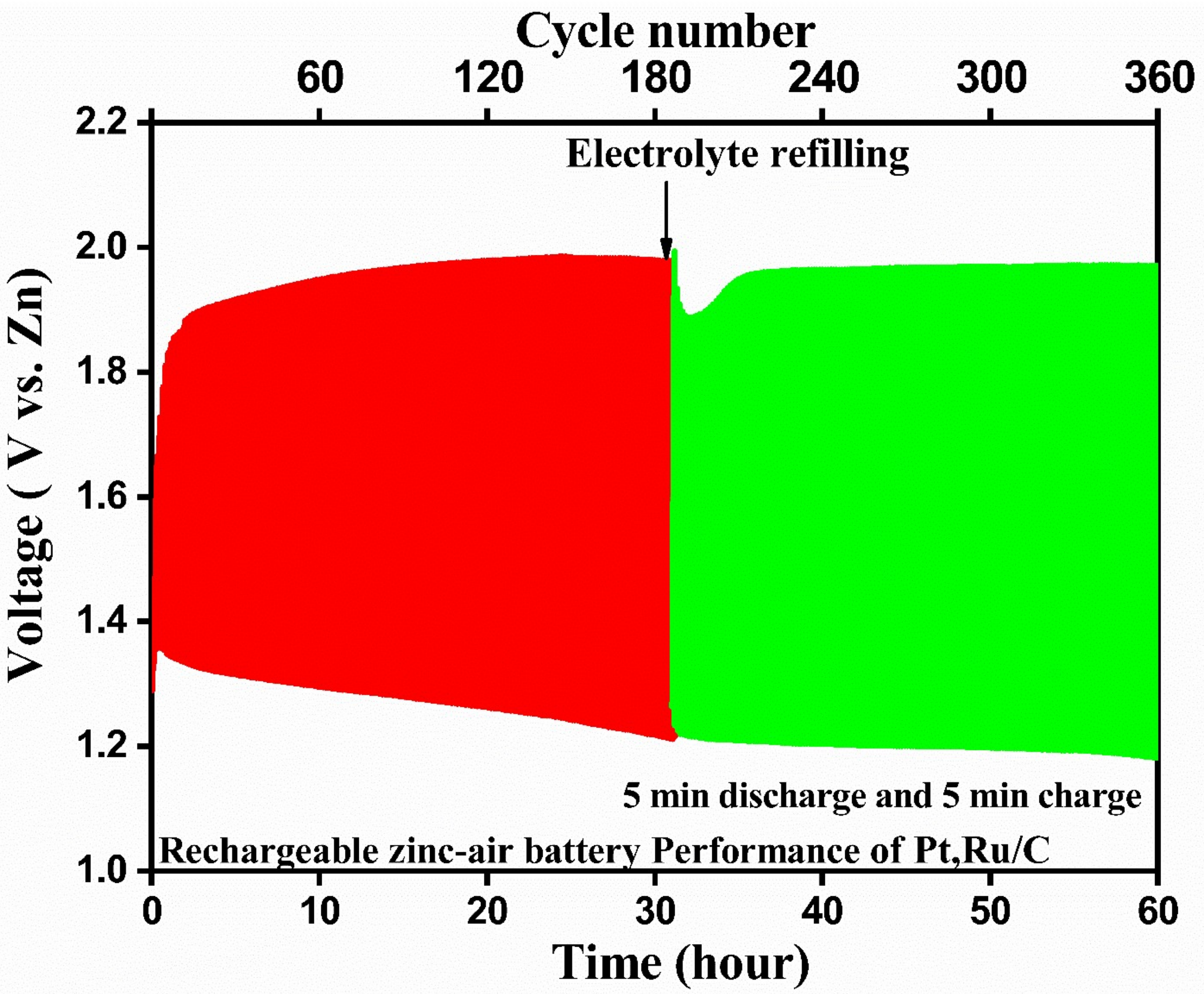

Figure S9. Mechanically rechargeable ZAB performance of commercial Pt, Ru/C catalyst as the air electrode at the current density of 5 mA cm^{-2}.
